# Temperature-Dependent Structural Changes of Parkinson's Alpha-Synuclein Reveal the Role of Pre-Existing Oligomers in Alpha-Synuclein Fibrillization

**DOI:** 10.1371/journal.pone.0053487

**Published:** 2013-01-22

**Authors:** Winny Ariesandi, Chi-Fon Chang, Tseng-Erh Chen, Yun-Ru Chen

**Affiliations:** 1 Genomics Research Center, Academia Sinica, Taipei, Taiwan; 2 Chemical Biology and Molecular Biophysics, Taiwan International Graduate Program, Academia Sinica, Taipei, Taiwan; 3 Department of Chemistry, National Tsing-Hua University, Hsin-Chu, Taiwan; University of Maryland School of Medicine, United States of America

## Abstract

Amyloid fibrils of α-synuclein are the main constituent of Lewy bodies deposited in substantial nigra of Parkinson's disease brains. α-Synuclein is an intrinsically disordered protein lacking compact secondary and tertiary structures. To enhance the understanding of its structure and function relationship, we utilized temperature treatment to study α-synuclein conformational changes and the subsequent effects. We found that after 1 hr of high temperature pretreatment, >80°C, α-synuclein fibrillization was significantly inhibited. However, the temperature melting coupled with circular dichroism spectra showed that α-synuclein was fully reversible and the NMR studies showed no observable structural changes of α-synuclein after 95°C treatment. By using cross-linking and analytical ultracentrifugation, rare amount of pre-existing α-synuclein oligomers were found to decrease after the high temperature treatment. In addition, a small portion of C-terminal truncation of α-synuclein also occurred. The reduction of pre-existing oligomers of α-synuclein may contribute to less seeding effect that retards the kinetics of amyloid fibrillization. Overall, our results showed that the pre-existing oligomeric species is a key factor contributing to α-synuclein fibrillization. Our results facilitate the understanding of α-synuclein fibrillization.

## Introduction

Intracellular aggregates of α-synuclein are the major pathological hallmark of Parkinson's disease, a progressive neurodegenerative disease due to dopaminergic neuron loss in the *substrantia nigra* [1]. PD is majorly idiopathic. Some cases were found in autosomal dominant PD that link to genetic point mutations in α-synuclein [2]. The fibrillar α-synuclein depositions are the primary substance found in Lewy bodies and Lewy neurites [1]. Physiological roles of α-synuclein have been shown to assist neurotransmitter release and vesicle recycling [3], maintain dopamine regulation through modulation of tyrosine hydroxylase activity and dopamine transporter shuttling [4], and block ER-Golgi trafficking [5]. In addition, extracellular seeding of α-synuclein aggregates are able to induce endogenous α-synuclein aggregation [6].

α-Synuclein with molecular mass of 14,460 Da comprises of 140 residues and possesses three structural regions. They are the N-terminal region with amphipathic helices containing a conserved 7 repetitive amino acids motif KTKEGV (residues 1–60), an amyloidogenic NAC (non-amyloid component) region (residues 61–95), and a C-terminal acidic tail region (residues 96–140). Recombinant α-synuclein monomer is a natively unfolded or intrinsically disordered protein (IDP) which lacks compact secondary and tertiary structures [7]. The disordered properties of α-synuclein have been examined by gel filtration, UV absorption, circular dichroism (CD), Fourier transform infrared spectroscopy (FTIR), Raman spectroscopy, small-angle X-ray scattering, and NMR [7]–[10]. In combination of paramagnetic relaxation enhancement (PRE) in NMR and molecular dynamic simulation, monomeric α-synuclein has been shown to possess long-range contacts between the C-terminus and NAC region that prevent its aggregation [11], [12]. The intramolecular contacts demonstrated that α-synuclein has slightly more compact structures rather than completely random coil contents [10]–[12]. Recently, endogenous α-synuclein isolated from red blood cells has been demonstrated to form a transient tetramer enriched in helical structures and is more resistant to aggregation [13]. However, other thorough analysis of the endogenous α-synuclein in mouse and rat brains as well as in different cell lines showed α-synuclein still existed as an unfolded monomer [14].

α-Synuclein is an amyloidogenic protein, which is prone to aggregate into specific cross-β amyloid fibrils as characterized by x-ray diffraction, Congo red birefringence, and fluorescence staining by thioflavin [15]. Amyloid formation plays an important role in neurodegenerative diseases. Many amyloid aggregates are considered the causative factors of the diseases, for examples, amyloid-β (Aβ) in Alzheimer's disease, scrapie form of prion in prion diseases, and polyQ expended proteins in Huntington disease and Ataxia [16]. Amyloid fibril is formed from a destabilized native structure or IDP that transformed into insoluble and highly ordered structure composed of β-sheets [17]. The kinetics of amyloid fibrillization is considered to adopt a nucleation dependent polymerization mechanism [18]. It starts from a rate-limiting nucleation phase to form oligomeric nucleus, followed by an elongation phase to grow fibrils, and finally reached a steady stage where the mature amyloid fibril formation are complete. The missense mutations found in PD patients corresponding to A53T, A30P, and E46K in the α-synuclein are more destabilized [19] and lead to faster aggregation [20], [21]. The intermediate oligomeric species of α-synuclein have been considered more toxic and correlate best with the disease [21], [22]. Therefore, the pathway of converting soluble α-synuclein to aggregates is thought to be crucial in PD etiology. Evidences have shown such conversion is triggered by both intrinsic properties, for example, genetic point mutations [20], [21], oxidation [23], and phosphorylation [24], and environmental factors, such as salts [25], pH [8], presence of seeds [26], and anionic lipids [27].

Understanding mechanisms of α-synuclein folding, oligomerization, and fibrillization is essential to potential therapeutic development against PD. To gain more insight on the native conformation and fibrillization mechanism of α-synuclein, here, we examined the effect of a short treatment of various temperatures on α-synuclein at the early stage and studied their conformational changes and the subsequent oligomerization and fibrillization. The recombinant α-synuclein was purified without heat denaturation to maintain its native conformation. We employed far-UV circular dichroism (CD) and NMR spectroscopy to study conformational changes of α-synuclein and thioflavin-T (ThT) fluorescence, transmission electron microscope (TEM), photo-induced cross-linking of unmodified proteins (PICUP), sedimentation velocity (SV) of analytical ultracentrifugation (AUC), and analytical size exclusion chromatography (SEC) to examine the effect on α-synuclein oligomerization and fibrillization.

## Materials and Methods

### Materials

Isopropyl β-D-1-thiogalactopyranoside (IPTG), Thioflavin-T (ThT), Bis-ANS (4,4′-Dianilino-1,1′-binaphthyl-5,5′-disulfonic acid dipotassium salt), Coomassie brilliant blue R-250, and Tris(2,2′-bipyridyl)dichlororuthenuum (II) (RuBpy) were purchased from Sigma-Aldrich. Ammonium persulfate was purchased from Merck. The 400-mesh Formvar carbon-coated copper grids were from EMS Inc., Uranyl acetate was from Structure Probe Inc., Q-sepharose FF column and Superdex G200 10/30 were both from GE. Trypsin with sequencing grade was from Roche and C18 Zip Tip from Millipore.

### Cloning, expression, and purification of α-synuclein

The human SNCA gene encoding α-synuclein was cloned from pOTb7 into pET21b using forward 5′CATTACATATGGATGTATTCATGAAAGGAC3′ and reversed 5′CTGTCAGCAGATCTCGAGAAACTGG 3′ primers creating XhoI and NdeI restriction sites. The α-synuclein coding sequence contained no extra residue and was confirmed by DNA sequencing using T7 sequencing primers. For protein expression, the construct was transformed into *E. coli* BL21 (DE3) and grew on an ampicillin plate. A single colony was inoculated, amplified, and grew to 1 L LB culture containing 50 µg/ml ampicillin with shaking at 250 rpm at 37°C. The protein expression was induced by adding 0.5 mM IPTG when OD600 reached 0.6. The cells were harvested 4 hr after induction and the protein was extracted following previous literature by periplasmic osmotic procedures without heating [28]. Briefly, the cell pellet was resuspended in 100 ml of 30 mM Tris-HCl buffer, pH 7.3, containing 40% sucrose. The solution was then centrifuged at 12,000 rpm for 20 min at 4°C. The sedimented pellet was further resuspended in 100 ml ice-cold water containing protease inhibitor and followed by the addition of 45 µl saturated MgCl_2_. The solution was incubated on ice for 10 min, followed by centrifugation at 12,000 rpm for 20 min at 4°C. The supernatant was then dialyzed into 25 mM Tris-HCl, pH 8 for purification on anion exchange chromatography.

To purify the protein, dialyzed α-synuclein was loaded onto a 5 ml Q-sepharose FF column and ran in 10 mM Tris-HCl buffer, pH 8 with a salt gradient from 100 to 500 mM NaCl. α-synuclein was eluted at approximately 225 mM NaCl. The elution fractions containing α-synuclein were confirmed by 15% SDS-PAGE and pooled together. The sample was further purified through spin filter with 30 kDa MWCO (Millipore, USA) to remove contaminants above 30 kDa by collecting the flow through. The flow through was concentrated by spin filter with 10 kDa cut-off to 200 µM and dialyzed by a 3-kDa cutoff dialysis membrane to 10 mM Tris-HCl, pH 7.4. The sample was filtered through 0.2 µm filter membrane (Millipore, USA) and stored at −80°C for the experiments.

### α-Synuclein preparation, temperature pretreatment, and fibrillization

α-Synuclein was thawed on ice and centrifuged at 17,000×g, 4°C, for 30 min to remove possible precipitants. After centrifugation, supernatant was collected and requantified by an absorbance spectrophotometer (DU800, Beckman Coulter) at 274 nm (ε = 5,960 cm^−1^ M^−1^ for full length α-synuclein and 1, 490 cm^−1^ M^−1^ for the C-terminal truncated one) [29]. Monomeric α-synuclein at 25 µM in 10 mM Tris-HCl, pH 7.4 was incubated in 5 different temperatures 20, 40, 60, 80, and 95°C simultaneously for 1 hr, and the samples were cooled back on ice and kept in room temperature before the experiments. The fibril formation assay was performed by addition of ThT and measuring the fluorescence with excitation at 444 nm and emission at 485 nm by an ELISA plate reader (M5, Molecular Devices, USA).The samples were incubated at room temperature with constant agitation at 1,000 rpm in an ELISA plate for ∼10 days.

### Far-UV CD spectroscopy

The CD spectra of α-synuclein were examined by J-815 spectrometer (Jasco) equipped with Peltier temperature cell holder controller. A 25 µM of α-synuclein was dissolved in 10 mM Tris-HCl, pH 7.4. Samples were pretreated with 5 different temperatures as indicated and subjected to far-UV CD. The solution was placed in a square quartz cuvette with 0.1 cm path length. The spectra were recorded with 1 nm band width and 1 nm step size. Buffer baselines were subtracted from the spectra. Thermal melting and cooling between 0 and 100°C were performed with a rate of 1.7°C/min with 1°C temperature increment. The signals were monitored at 222 nm. The data were normalized and plotted as molar ellipticity (deg M^−1^ cm^−1^).

### TEM

The morphology of the end-point products from α-synuclein fibrillization studies was visualized under TEM. The 10 µl samples were spotted on glow-discharged, 400-mesh Formvar carbon-coated copper grids for 5 min, washed twice by double distilled water, and negatively stained with 2% filtered uranyl acetate. The images were captured by Tecnai G2 Spirit Twin TEM (FEI, Hillsboro, OR, USA) with an accelerating voltage of 75 kV.

### PICUP

PICUP assay was performed following previous literature [30], [31]. The samples were subjected to PICUP immediately after temperature treatment of α-synuclein to monitor the oligomeric species. Briefly, a volume of 18 µl α-synuclein was mixed with 1 µl of 0.5 mM RuBpy and 1 µl of 1 mM ammonium persulfate and followed by irradiation with a blue light LED for 1 s. The reaction was then quenched by addition of 10 µl SDS-PAGE sample buffer containing 5% β-mercaptoethanol. The samples were loaded in 16% Tris-tricine SDS-PAGE gel and proceed for western blotting using the primary antibody anti-syn211 (1∶20,000) recognizing α-synuclein residues 121–125 (Chemicon) and the secondary antibody goat anti-mouse HRP conjugated IgG (1∶10,000).

### ^1^H^15^N HSQC and dynamic motion of α-synuclein

NMR measurements were performed by using 200 µM of ^15^N-labeled α-synuclein dissolved in 10 mM sodium phosphate buffer, pH 7.4, in H_2_O/D_2_O (9∶1, v/v). The ^15^N-labeled α-synuclein was expressed in the M9 minimal media containing ^15^NH_4_Cl and purified as described for the unlabeled protein. NMR spectra were acquired at 10°C on Bruker Avance 600 MHz NMR spectrometer equipped with 5 mm triple resonance cryoprobe and Z-gradient. Amide ^15^N-^1^H chemical shifts were assigned based on previous deposited data in the Biological Magnetic Resonance Data Bank (BRMB number 16543) on α-synuclein [32]. The T2 spectra were measured at 16, 49, 82, 98, 131, 196, and 228 ms. The transverse relaxation rates (R2) for backbone amides were then obtained by the two parameter nonlinear optimization of a single exponential function using the software Protein Dynamics Center (Bruker Biospin).

### AUC

Sedimentation velocity (SV) of AUC experiments were performed in a Beckman Optima XL-1 analytical ultracentrifugation. The experiments were carried out with 25 µM α-synuclein in 10 mM Tris-HCl, pH 7.4, with and without temperature treatment at 95°C for 1 hr. The experiments were performed at 42,000 rpm for 45 hr at 4°C using an An-60Ti rotor (Beckman) and the moving boundary was monitored by the absorption at 230 nm. The data were analyzed by SEDFIT (U.S. NIH) and the parameters were calculated using SEDNTERP (NIH).

### SEC

α-Synuclein at 100 µM with and without temperature treatment at 95°C for 1 hr were subjected to SEC. The samples were centrifuged at 17,000×g, 4°C, for 30 min. The supernatant was loaded onto a Superdex G200 10/30 gel filtration column in 10 mM Tris-HCl, pH 7.4 with a flow rate of 0.4 ml/min. The protein absorption at 280 nm was monitored. Molecular weight standards including albumin (67 kDa), ovalbumin (43 kDa), and RNase (13.7 kDa) were ran under the same condition and indicated. To isolate the truncated form of α-synuclein, we prolonged the heat treatment for 6 hr at 80°C. The peak fractions were pooled and subjected to PICUP assay and the cross-linked species were detected by western blotting following previous protocol.

### Mass spectrometry

The MALDI-TOF measurement was performed with Ultraflex II MALDI-TOF/TOF mass spectrometer (Bruker) equipped with a nitrogen pulsed laser (355 nm). Tryptic digested bovine serum albumin (1 pmol/µL) was mixed with 2,5-dihydroxybenzoic acid (DHB) (10 mg in 1∶1 acetonitrile/water and 0.1% trifluoroacetic acid) as the standard for MALDI-TOF mass calibration. The α-synuclein sample and matrix mixture was spotted onto a MALDI plate and the data points were collected at the average of 500 shots of the laser beam with laser fluence ∼30%. Mass spectra were obtained in the range of mass to charge ratio (*m*/*z*) from 1,000 to 10,000. The spectra were analyzed by using Bruker Data Analysis software. The in-gel digestion and LC-MS/MS experiments were performed after cut out the desired bands with a surgeon blade. The gel pieces were soaked by the destaining solution containing 25 mM NH_4_HCO_3_/50% acetonitrile (ACN) and dried by vacuum. The dry gel piece were incubated with a minimum tryptic solution volume, 10–20 µl with trypsin concentration at 12.5 ng/µl, at 4°C for 10 min for rehydration. Then, the digestion was performed at 37°C overnight. The samples were washed with 100% ACN and loaded to C18 Zip Tip for extraction. Digestion were stopped by 50% ACN/0.1% TFA The peptides were desalted and eluted in 60% ACN/0.1% TFA. The eluents were subjected to LC-MS/MS analysis. The LC-MS/MS experiments were performed on a linear quadrupole ion trap Fourier transform ion cyclotron resonance (LTQ-FT Ultra) mass spectrometer (Thermo Electron) equipped with a nanoelectrospray ion source (New Objective), an Agilent 1100 Series binary high performance liquid chromatography pump (Agilent Technologies), and a Famos autosampler (LC Packings). The procedure was performed as described previously [33]. Briefly, the peptide mixtures were separated by a self-packed reversed phase C18 nanocolumn. Electrospray voltage was applied at 1.6±0.2 kV, and capillary temperature was set at 200°C. MS/MS spectra were collected in the LTQ mass spectrometer and the peptide assignments were processed by Mascot Daemon software (version 2.2, Matrix Science).

## Results

### High temperature treatment inhibits α-synuclein fibril formation

**Figure 1:**
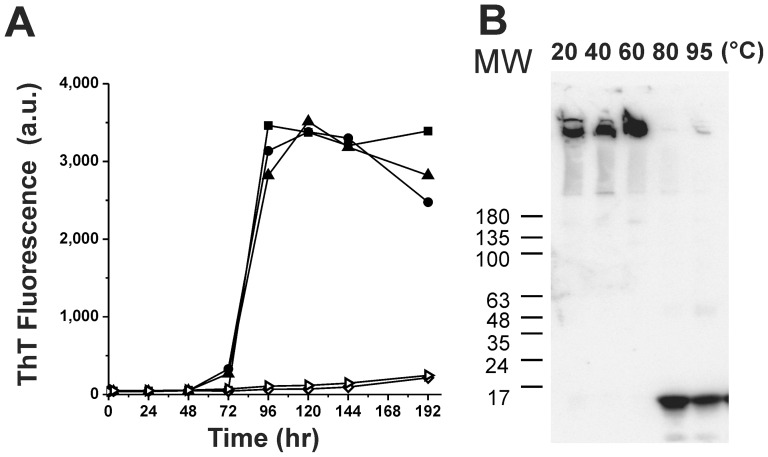
High temperature pretreatment inhibits α-synuclein fibril formation. (A) α-Synuclein (25 µM) was subjected to 1 hr pretreatment in 20 (•), 40 (▴), 60 (▪), 80 (⋄), and 95 (▹) °C and the fibril formation was monitored at room temperature by addition of 20 µM ThT with continuous shaking at 1,000 rpm for 10 days. (B) Western blotting of the end-point products of α-synuclein in ThT assay. The pretreated temperatures and the molecular weight markers are indicated.

To understand the relationship between the native conformation of the intrinsically disordered α-synuclein and its amyloid fibril formation, we utilized an environmental denaturing factor, high temperature, to study the temperature effect on its fibrillization process. To retain the native conformation of α-synuclein, we purified the protein via an osmotic shock procedure [28], [34] without using the heating method [7], [35]. The recombinant α-synuclein purified was predominantly a monomeric species as evidenced by later examinations of AUC. The random coiled property shown by far-UV CD was similar to the unfolded monomer of α-synuclein reported [14] but different from the spectra of tetrameric α-synuclein with helical contents [13], [36]. We treated the freshly prepared α-synuclein at various temperatures using water baths at 20, 40, 60, 80, and 95°C simultaneously and let them cooled back to the room temperature and subjected to fibrillization using ThT assay. ThT is a fluorescence amyloid probe that can specifically bind to the cross-β structure of amyloid fibril spine [37]. The fibrillization process was monitored for more than 192 hr with continuous shaking. For the temperature treatments at 20, 40, and 60°C, we found that α-synuclein formed fibrils following a classic amyloid fibril formation pattern. It showed a lag time for ∼72 hr, an elongation from 72 to 96 hr, and a steady stage from 96 to 192 hr. However, interestingly the fibril formation process showed a dramatic reduction and inhibition after 1 hr pretreatment of α-synuclein at 80 and 95°C. The ThT level at the steady state was more than 10 fold lower than the others after lower temperature pretreatments (Figure 1A). The result showed the high temperature pretreatment significantly affected α-synuclein fibrillization.

**Figure 2:**
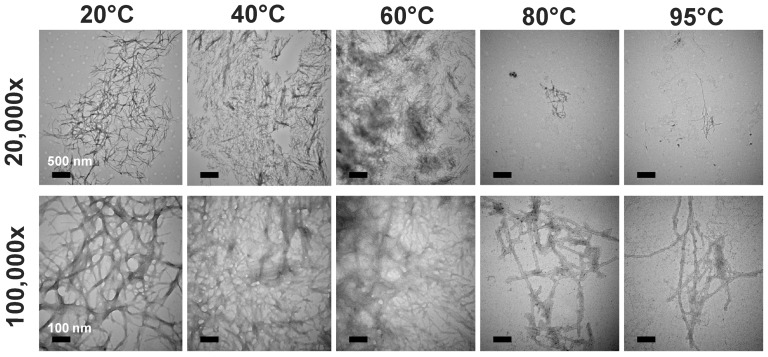
Fewer and less crowded α-synuclein fibrils were formed after high temperature pretreatments. The fibril morphology of α-synuclein after incubation was examined by TEM at 20,000× (scale bar = 500 nm) and 100,000× (scale bar = 100 nm) magnifications. The pretreated temperatures are indicated.

Since ThT signal is not quantitative and may be affected by the binding environment, we confirmed the results by western blotting and TEM imaging. We subjected the end-point products from the fibril formation assay to western blotting. We found large amount of high-molecular-weight aggregates of α-synuclein detected in the stacking gel for the treatments at 20, 40 and 60°C. However, the large aggregates of α-synuclein almost diminished while the samples were pre-treated with high temperature at 80 and 95°C. The monomeric species that migrated at ∼17 kDa were dominant after such pre-treatments (Figure 1B). By examining the morphology of the end-point products of fibril formation by TEM images (Figure 2) we found clusters of mass amount of fibrillar tangles with the 20, 40 and 60°C. In contrast, rare fibrils were found in the samples pretreated with 80 and 95°C. The imaged fibrils found were less tangled. The TEM results were consistent with the ThT assay and western blotting. Therefore, we found that α-synuclein pretreated with 20, 40, and 60°C formed similar degree of fibrils but after high temperature pretreatment few fibrils were found.

### The secondary structures of α-synuclein are fully reversible in thermal melting

**Figure 3:**
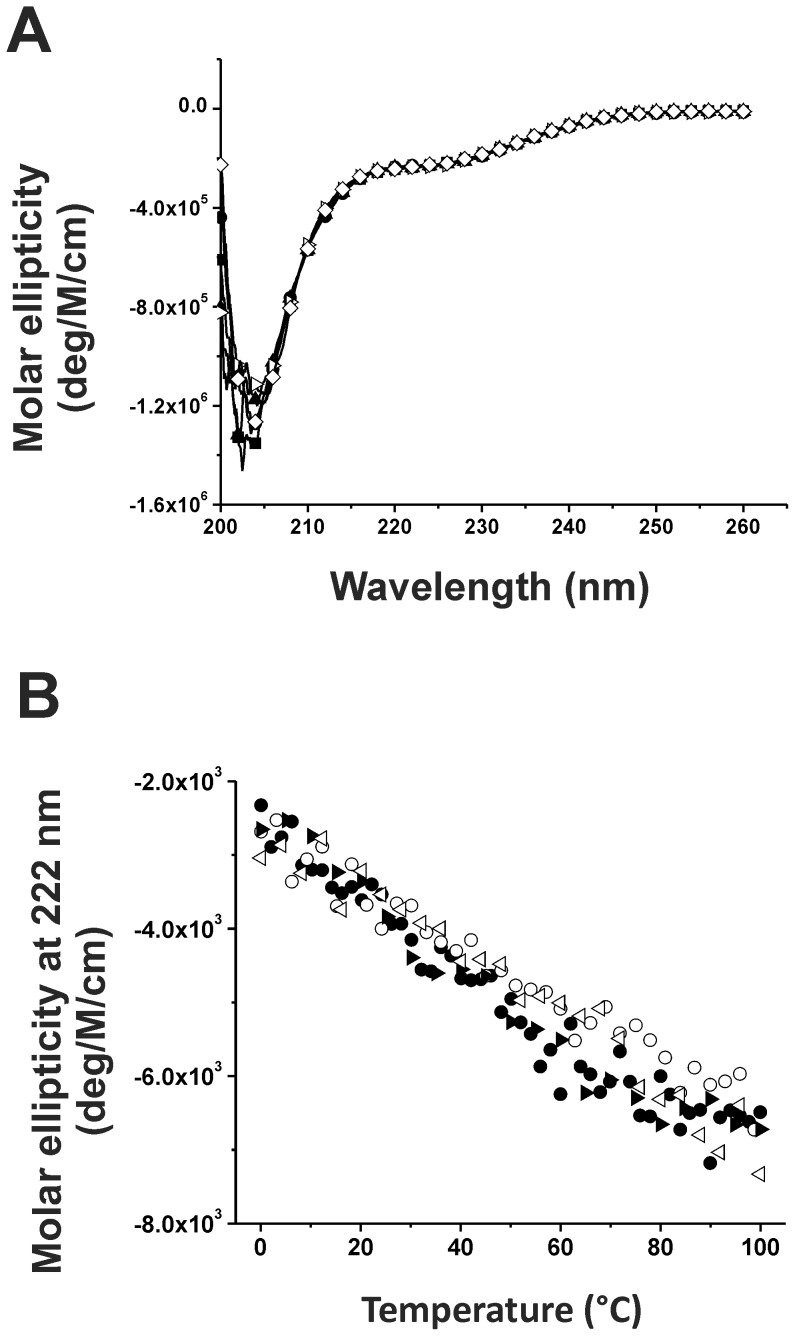
Secondary structures of α-synuclein retained after temperature pretreatments and were fully reversible upon temperature denaturation. (A) Conformational changes of α-synuclein at 25 µM after pretreatment in 5 different temperatures, 20 (•), 40 (▴), 60 (▪), 80 (⋄), and 95 (▹) °C, for an hr were examined by far-UV CD spectra at room temperature. The data showed α-synuclein adopted random-coiled like structures without conformational changes after the temperature pretreatments. (B) Thermal melting and cooling of 25 µM α-synuclein treated with 20 (•,○) or 95°C (▸,▹). The temperature was increased from 5 to 100°C (solid symbols) or decreased from 100 to 5°C (empty symbols) with temperature changed at a rate of 1.7°C/min. The CD signals at 222 nm were monitored and the molar ellipticity is plotted. The data showed a completely reversible folding of α-synuclein in response to temperature. No protein loss was found in the experiment.

We next asked the question whether the native conformation of α-synuclein was affected by the temperature treatment that leads to the differences shown in the fibrillization. We first measured the absorption spectra before and after the temperature treatment. After the treatment, the samples were centrifuged and the supernatant was subjected again to absorption measurement. The spectra were fully identical indicating no protein loss occurred after treatment in five different temperatures (data not shown).We employed far-UV CD spectra to examine the possible conformational change of α-synuclein (Figure 3A). The far-UV CD spectra showed that α-synuclein at 20°C has predominantly random-coil structures as expected. However, the CD spectra after the various temperature pre-treatments showed literally no change indicating that their secondary structures were not affected upon temperature treatments. We further examined the spectra changes by thermal melting and cooling (Figure 3B). The far-UV CD spectra of α-synuclein were recorded from 0°C and consecutively heated to 100°C then cooled back to 0°C at a rate of 1.7°C/min. The signals at 222 nm obtained from both melting and cooling were plotted against temperature. The individual sample showed complete reversible pattern for heating and cooling and the different temperature pretreatments did not result in significant difference in the heating and cooling pattern. The result showed that α-synuclein possesses conformational plasticity that is fully reversible upon temperature denaturation.

### No atomic level conformational changes from NMR observation

**Figure 4:**
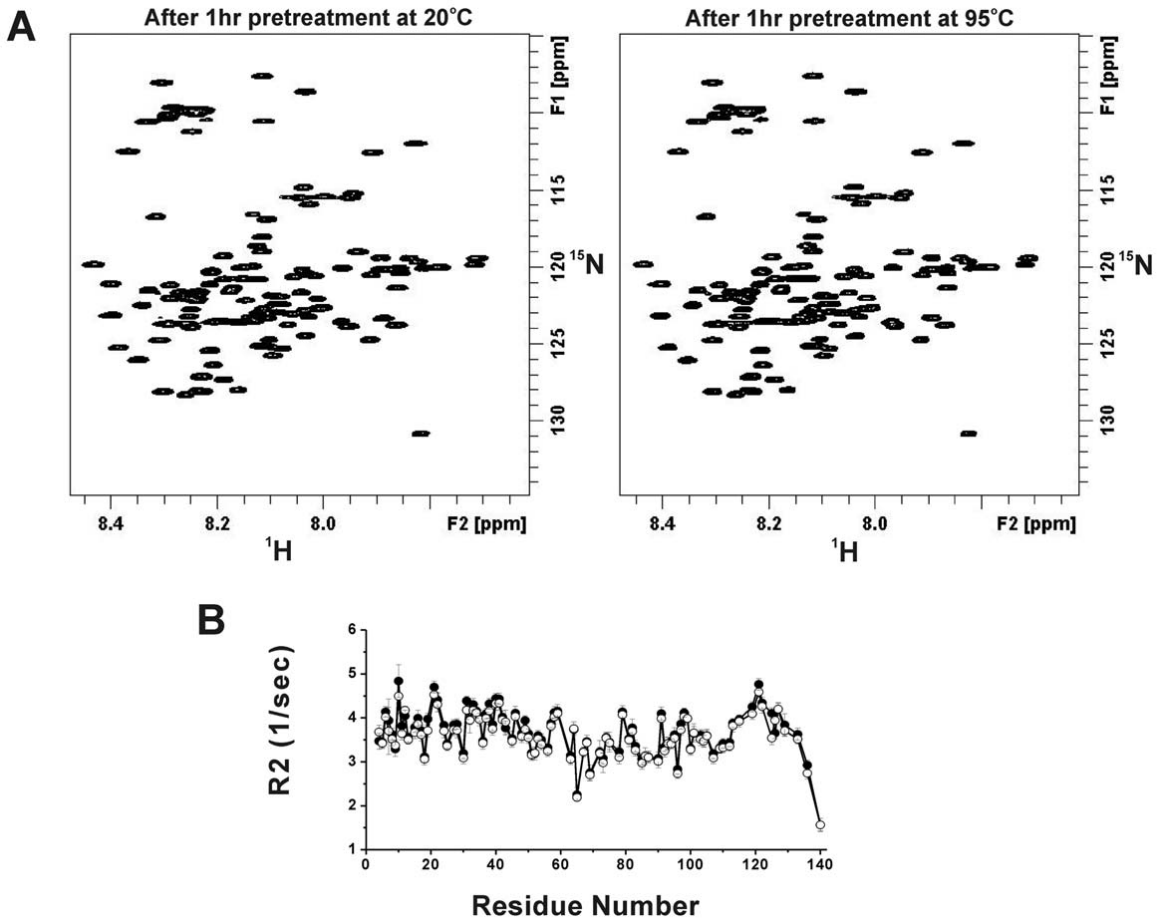
^1^H-^15^N HSQC NMR spectra and NMR dynamics of α-synuclein showed no significant difference in chemical shifts and relaxation rates after the temperature pretreatments. (A) The ^1^H-^15^N HSQC spectra of 200 µM ^15^N-labeled α-synuclein in 10 mM phosphate buffer, pH 7.4 after 1 hr pretreatment at 20 or 95°C. All spectra were obtained at 10°C. (B) R2 relaxation rate of α-synuclein with (○) and without (•) pretreatment at 80°C for 6 hr. All spectra were obtained at 10°C.

To confirm the result from far-UV CD, we employed NMR techniques to observe the atomic level information of the tertiary structure of α-synuclein. The two-dimensional ^1^H-^15^N hetero nuclear single quantum coherence (HSQC) spectra of ^15^N labeled α-synuclein was examined after five temperature pretreatments and the chemical shifts were monitored at 10°C. The HSQC spectra were assigned based on previous study [32]. We found no significant changes in chemical shifts after 20 and 95°C temperature treatments (Figure 4A) indicating no conformational changes could be detected. We further examine the backbone spin-spin relaxation rate (R_2_) of α-synuclein which is sensitive to protein dynamics. Our relaxation data showed no significant difference between the sample with and without 80°C pre-treatment (Figure 4B); however, the average R_2_ value reduce a little after 80°C pre-treatment suggesting the heat treatment may cause the average molecular tumbling rate slightly faster. Overall, our NMR results indicated that there is no conformational change with and without the high temperature pretreatment or the changes were minor and insensitive to these techniques. These results were consistent with the CD observation on the reversibility of α-synuclein in thermal melting and cooling. Therefore, the intrinsic conformation of α-synuclein is unlikely to be responsible for the temperature effect on α-synuclein fibrillization.

### α-Synuclein oligomers were reduced after elevated temperature pretreatments

**Figure 5:**
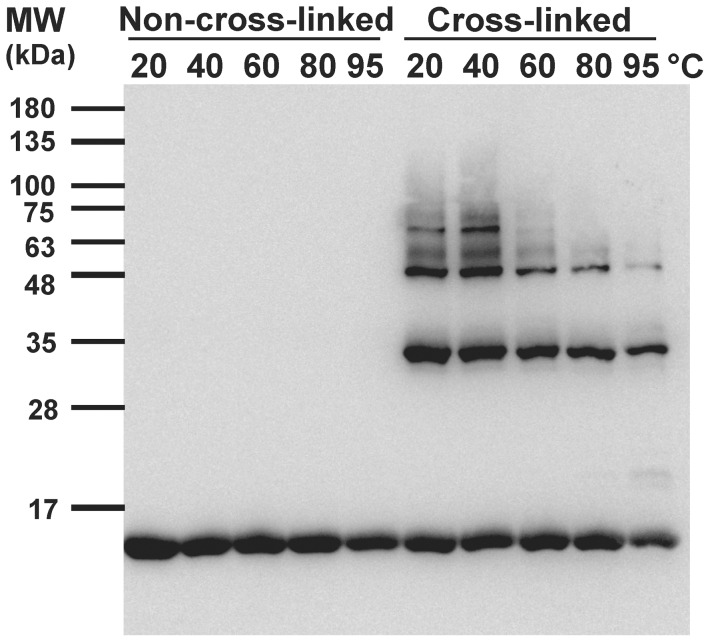
Transient oligomers existed in the freshly prepared α-synuclein. α-Synuclein after pretreatment from 5 different temperatures for an hr were subjected with and without PICUP and western blotting using anti-synuclein antibody, anti-syn211. Metastable oligomers including dimers, trimers, tetramers were observed after PICUP and the amount of oligomers were reduced after higher temperature pretreatments.

To further investigate the possible factors that result in retarding fibrillization after high temperature treatment, we characterized the early assembly of α-synuclein after different temperature treatment by PICUP assay (Figure 5). PICUP assay facilitates the covalent cross-linking of transient oligomeric species in the samples after photo stimulation. The technique allows the rare oligomeric species to become detectable which has been successfully demonstrated in accessing transient Aβ and α-synuclein oligomers presented in the early state [30], [31], . In the western blotting, α-synuclein without PICUP migrated below 17 kDa as a monomeric species, whereas, after PICUP multiple oligomers were detected. The oligomeric species shown on the gel migrated as dimers (∼34 kDa), trimers (∼51 kDa), and tetramers (∼68 kDa). The amounts of oligomers, especially tetramers and trimers, were found to be decreased after heat treatment >60°C. The tetrameric species were completely disappeared in higher temperatures and the trimer was much reduced in 95°C treated sample. Our data suggested that some proportion of pre-existing oligomers were affected by the heat treatment.

**Figure 6:**
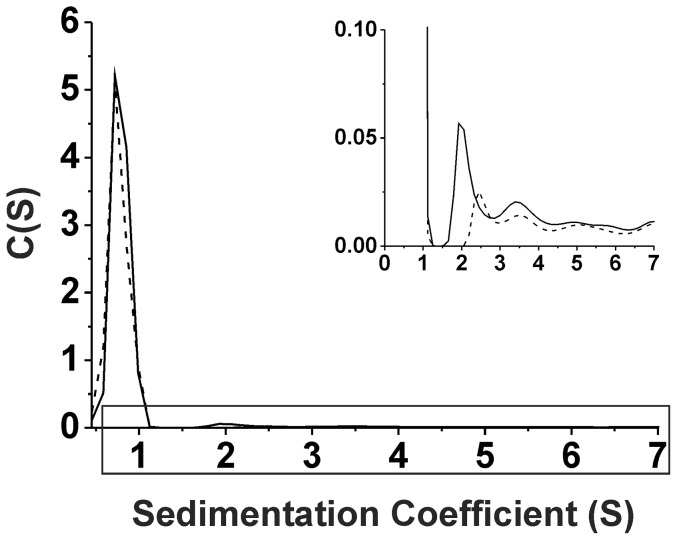
The preexisted α-synuclein oligomers were disrupted after 95°C treatment. α-Synuclein with (dash line) and without (solid line) 95°C treatment were analyzed by AUC-SV and the c(S) distribution is plotted against the sedimentation coefficient ranging from 0 to 7 S. The inset is the enlarged region from c(S) signal below 0.1.

In addition, we employed sedimentation velocity (SV) technique of AUC to validate the possible pre-existence of the oligomers (Figure 6). SV-AUC is able to provide species distribution of α-synuclein in the native samples and enables us to quantify the assembly based on their sedimentation properties. α-Synuclein samples were pretreated with and without 95°C for 1 hr and ran at 42,000 rpm, 4°C overnight. For the sample without the heat treatment, α-synuclein predominantly existed as a monomer situated at 0.7 S. The monomeric species was account for ∼94% of the total species detected. The friction ratio was fitted to 1.9 indicating α-synuclein monomer is a rod-shape protein. After zooming into higher sedimentation coefficient regions, several minor peaks situated at a range of 1.5 to 7 S were observed (Figure 6, inlet). These peaks represented the oligomers existed in the sample. After 95°C treatment, we also observed a monomer peak at 0.7 S, ∼96%, with similar intensity like that for the one without heat, but there was less amount of oligomers situated at a range of 1.5 to 7 S. Further analysis using MW calculation in SEDFIT, the species were assigned to the possible assembly as described in Table 1 and 2.

**Table 1:** Sedimentation coefficients showed species distribution of α-synuclein without heating.

Sedimentation Coefficient (S)	Relative Abundance (%)	Estimated MW (kDa)	Estimated Oligomeric State
0.7	93.8	16.6	Monomer
1.9	2.9	77.9	Tetramer/Pentamer
3.4	1	135	Octamer
4.9	1.3	244	Large oligomer
7.0	0.9	444	Large oligomer

**After 1 hr pretreatment at 20°C.**

Estimated molecular masses are calculated based on continuous c(S) distribution model (SEDFIT) and the corresponding state of oligomerization are also indicated based on sedimentation coeficient (S) value.

**Table 2:** Sedimentation coefficients showed species distribution of α-synuclein pretreated with heating.

Sedimentation Coefficient (S)	Relative Abundance (%)	Estimated MW (kDa)	Estimated Oligomeric State
0.7	95.6	15.5	Monomer
2.5	1.5	99.1	Tetramer/Pentamer
3.5	0.8	163	>Octamer
5.0	1.1	283	Large oligomer
7.0	1	449	Large oligomer

**After 1 hr pretreatment at 95°C.**

Estimated molecular masses are calculated based on continuous c(S) distribution model (SEDFIT) and the corresponding state of oligomerization are also indicated based on sedimentation coeficient (S) value.

### High temperature treatment induces a minor C-terminal truncated α-synuclein species

**Figure 7:**
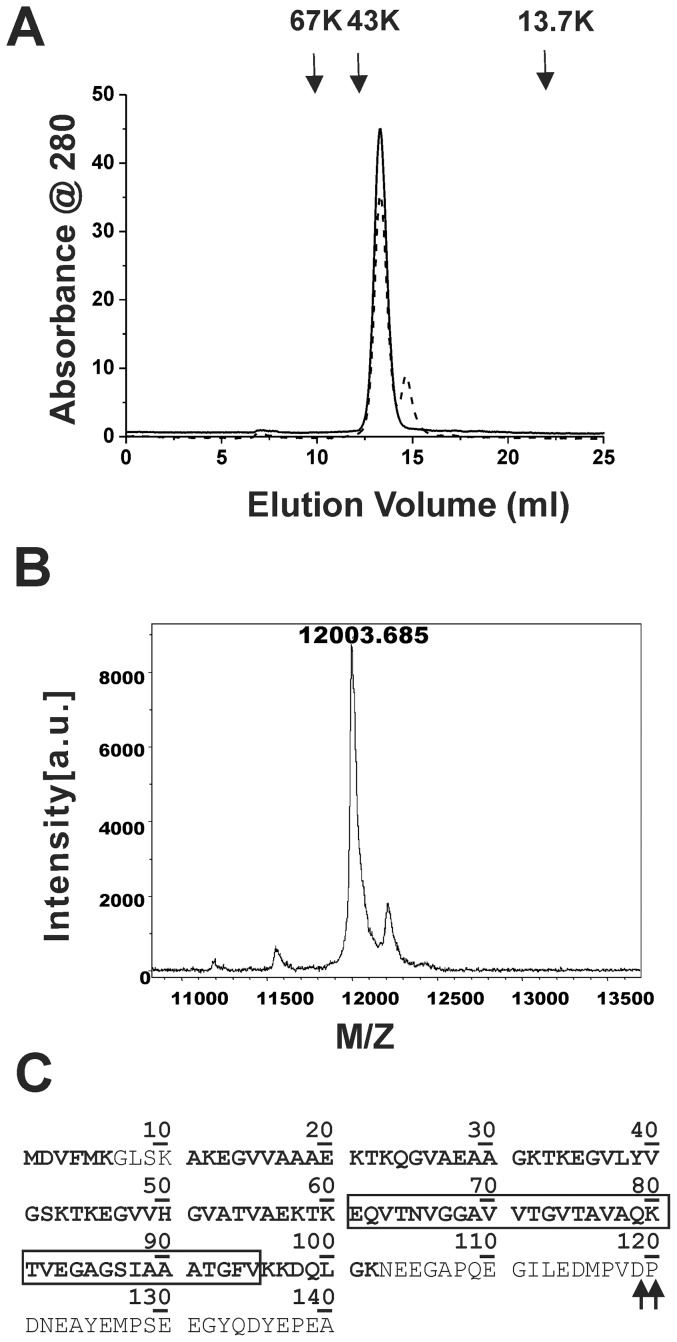
C-terminal truncation was induced after high temperature treatment. (A) α-Synuclein after treatment at 20 (solid line) and 95°C (dash line) for 1 hr were subjected to analytical SEC. The signal was monitored at absorbance 280 nm. The molecular weight markers are indicated. (B) The truncated species observed after heat treatment was characterized to have a molecular mass of 12,004 kDa by MALDI-TOF mass spectrometry. (C) The sequence identified from LC/MS/MS of the truncated species matched to the N-terminal and NAC regions of α-synuclein. The detected residues are labeled in bold and the arrow heads indicate the predicted truncation sites. The NAC region is boxed.

To further investigate the presence of α-synuclein oligomers, we try to separate and collect α-synuclein species by analytical SEC (Figure 7A) based on their hydrodynamic radius. By comparing the two samples pretreated with 20 or 95°C, we found the major peaks of the two samples were both eluted at ∼13.3 ml. The 95°C treated sample had slightly lower intensity, 35 mAU, than the 20°C treated one, 45 mAU. The decrease was approximately 19%. Comparing to the molecular weight standards, the peak was slightly smaller than 43 kDa. Since α-synuclein has been shown to elute at a higher molecular weight region due to its non-globular, extended conformation [7], the peak was considered the major monomeric species and later confirmed by MALDI/TOF. We did not detect any significant oligomeric species that should be eluted earlier than the major peak in SEC. This can be due to the rare amount of pre-existed oligomer species. However, interestingly in the 95°C treated sample, a minor peak eluted at 14.8 ml with one fifth intensity of the major peak was observed.

**Figure 8:**
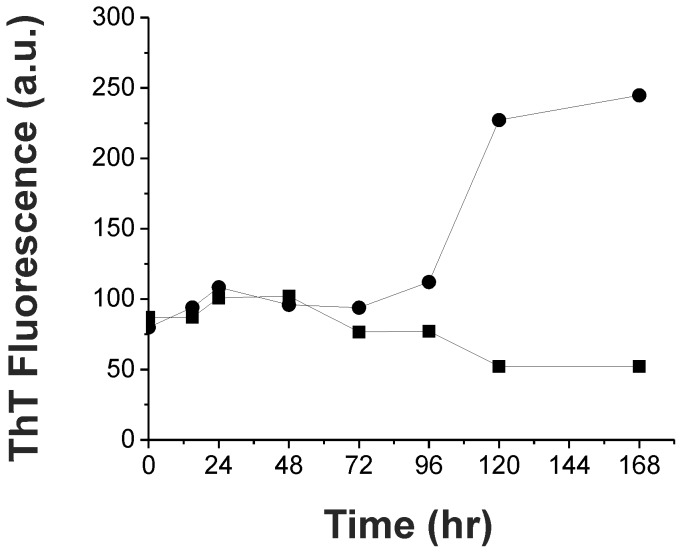
C-terminal truncated α-synuclein does not undergo fibrillization. Fibrillization assay of the isolated full-length (•) and C-terminal truncated (▪) α-synuclein. The fibrillization process was monitored by ThT fluorescence.

We further examine the minor peak found only in the sample after 95°C treatment by MALDI/TOF (Figure 7B). The molecular mass of the fraction determined was 12,003 Da indicating a possible smaller species in such fraction. Since the species was not detected in western blotting using antibody recognizing α-synuclein at C-terminal residues 121–126, we further confirm the identify of the species by LTQ-FT-MS. We treated α-synuclein in 95°C for an hr, isolated the truncated band migrated at ∼12 kDa in SDS-PAGE, and subjected that to in-gel tryptic digestion. The peptide fragments were sequenced by LTQ-FT-MS and the identified residues matched to the primary sequence of α-synuclein (Figure 7C, residues identified was shown in bold). We identified nearly all residues in the N-terminal and NAC regions. However, the last ∼38 residues at the C-terminal were missing due to lack of trypsin cleavage sites. Based on sequence prediction, we estimated the truncated site induced by high temperature treatment at either Asp119 or Pro120. To further investigate the effect of C-terminal truncation, we subjected the full-length and truncated α-synuclein isolated from SEC to fibrillization assay. The proteins at final concentration of 25 µM were re-quantified based on their extinction coefficients. The result showed that the C-terminal truncated α-synuclein was unable to form fibril (Figure 8) in our experimental time frame, whereas the full-length α-synuclein underwent fibrillization as expected.

## Discussion

The intrinsic disordered Parkinson's α-synuclein is an important protein of interest due to its disease relationship as well as structural categorization as an IDP. The intrinsic property of IDPs like α-synuclein and Alzheimer's Aβ are difficult to characterize since they lack of intact secondary and tertiary structures. By applying agents to influence IDP's properties can facilitate the understanding of the molecular mechanism. For example, in response to the supercooled and high hydrostatic pressure conditions, the α-synuclein fibrils have been found to rapidly dissociate [39], [40]and the hydrophobic and electrostatic interactions in the monomer are attenuated at −15°C [39]. Here, we used multiple biochemical and biophysical methods to examine α-synuclein conformation and aggregation in response to temperature treatment and ask whether its residual structures contribute to the fibrillization process. According to our far-UV CD and NMR results, α-synuclein monomer structure and dynamics were not significantly altered, if any, with and without the temperature treatment. The thermal melting experiment also showed a fully reversible behavior. The data suggested that thermal stress did not denature the residual conformation of α-synuclein which is consistent with some other IDPs [41], however, an increase of α-helical content [42] and loss of PPII structure upon increasing temperature have been reported [43]. The stability of α-synuclein monomer has been suggested as a major factor that affects the nucleation phase in fibrillization process. The native residual α-synuclein conformation was shown to possess long-range contacts between C-terminal and NAC regions that prevent aggregation by paramagnetic relaxation enhancement (PRE) NMR spectroscopy and molecular dynamic simulation [44]. The intramolecular contact shows that the native α-synuclein conformation has slightly more compact structures rather than completely random coils [11]. Although we did not performed PRE study, we found the R2 relaxations with the high temperature treatment are slightly smaller which may be due to the reduction of oligomer formation as observed from AUC and PICUP assays. We also compared our ESI/LC/MS results of α-synuclein with and without heating to examine the possible heat-induced oxidative modifications (Figure S1). The results showed that although there were oxidized α-synuclein existing (mass peaks at ∼14467 and ∼14481 Da), the intensities of the oxidized peaks in the heated and non-heated samples have no difference. There were no new modification occurred due to heat treatment. Therefore, we conclude that our heating procedure did not induce further modifications.

α-Synuclein oligomers are believed to play a major role in neuronal dysfunction and loss in Parkinson's disease [45]. The species appeared in the fibrillization pathway then convert to mature fibrils. Amyloid seeding effect is a common phenomenon in the amyloidogenic proteins. It has been used to generate homogeneous fibrils for structural characterization and amplification [46] or to eliminate the nucleation step to form amyloid oligomers [47], [48]. Recently, oligomer cross-seeding among different amyloid proteins, Aβ and tau, have also been shown [49]. In our study, we observed changes in the population of α-synuclein oligomers with and without the temperature treatment. By photo-crosslinking the freshly prepared α-synuclein, the short-lives metastable oligomers were enhanced and we found reduced population of α-synuclein tetramer, trimer, and dimers after high temperature treatment. The data were consistent with the AUC-SV study showing reduction of oligomer species. However, the species estimated from AUC-SV analysis were larger than those seen on SDS-PAGE after PICUP. The assembly difference should be due to the denaturing SDS environment for PICUP species since we have observed similar phenomenon in Aβ oligomer size reduction when comparing the SEC data and PICUP study (unpublished data). Our study showed that the recombinant α-synuclein contains a trace amount of oligomer seeds. We have also used lyophilized α-synuclein with and without hexafluoroisopropanol (HFIP) treatment to study the oligomer appearance and its relationship to fibrillization (Figure S2), we found HFIP treated samples had retarded fibrillization (Figure S2A) and possessed less oligomer species (Figure S2B). The result is consistent with our finding in the heat treatment. Since the percentage of seeds populated in the solution plays a critical role for accelerating the fibrillization kinetics via elimination of the nucleation phase [50], reduction of pre-existing oligomer seeds should contribute to retardation of the fibrillization process after high temperature treatments.

The negatively charged C-terminal tail of α-synuclein is involved in the regulation of aggregation possibly via intramolecular contacts to stabilize non-fibrillar monomers [11], [12], [51]–[54]. The C-terminal truncated forms, amino acids (a.a.) 1–108 and a.a.1–124 were shown to aggregate faster than the full-length α-synuclein [51] and a.a. 1–102, 1–110, 1–120, 1–130 have been shown to form higher aggregates in the turbidity assay, although the a.a. 1–120 and 1–130 form shorter fibrils [54]. By employing proline to alanine mutants in the five proline residues on the C-terminal tail, it has been shown that most of the proline mutants accelerate the fibrillization, but the proline (120, 128, 138) to alanine mutants retards the kinetics [55]. Our results showed that the C-terminal truncation occurred to ∼19% of the monomer after 95°C pretreatment, which has a much slower fibrillization process. In addition, the re-quantified C-terminal truncated α-synuclein, likely residues 1–120, did not aggregate into ThT positive fibrils in our experimental time frame, whereas the full-length did. The discrepancy can be due to the difference in the protein purification methods and the experimental conditions. We also speculated the lost of proline residues 120, 128, and 138 maybe responsible for the fibril retardation. Furthermore, previous study on co-assembly of full-length and truncated α-synuclein showed that a.a. 1–120 is incompetent to seed full-length α-synuclein whereas the a.a. 1–102 and 1–110 are competent [54]. This may indicates the possible loss of seeding effect of the monomer besides the oligomer reduction after the high temperature treatment. However, it is also likely that the C-terminal truncation occurred as a minor coincidence of peptide hydrolysis after heating that may not contribute majorly to the fibril retardation.

## Conclusion

Our study demonstrated that α-synuclein fibril formation was substantially inhibited after high temperature pretreatment that are likely due to diminishing of preformed oligomeric seeds and C-terminal truncation rather than altering the residual monomer conformation. Overall, our results facilitate understanding of the role of oligomer seeds and C-terminal of α-synuclein in its amyloid fibril formation.

## Supporting Information

Figure S1 **Nano-LC-ESI-MS results showed oxidative modifications of α-synuclein were not affected by the high temperature treatment.** (**A**) α-Synuclein without heat treatment and (**B**) after treatment at 80°C for 6 hr.
(JPG)

Figure S2 **HFIP pretreatment inhibits α-synuclein fibril formation.** (**A**) α-Synuclein samples were pretreated with (▪) and without (•) HFIP to remove pre-aggregates and dried by speed vacuum to remove the solvent. α-Synuclein was dissolved in 20 mM Tris-HCl, pH 7.4, centrifuged at 17,000×g at 4°C for 30 min and incubated in continuous shaking for 7 days. (**B**) PICUP assay showed α-synuclein dimer and trimer were reduced after HFIP treatment. The western blot was probed by anti-syn 211 antibody.
(JPG)

Method S1 **Temperature effect on Parkinson's α-synuclein.**
(PDF)
