# Supplementary figures and images for: Temperature-Dependent Structural Changes of Parkinson's Alpha-Synuclein Reveal the Role of Pre-Existing Oligomers in Alpha-Synuclein Fibrillization
Source: PLoS One. 2013 Jan 22;8(1):e53487. doi: 10.1371/journal.pone.0053487 (PMC3551866; doi:10.1371/journal.pone.0053487)

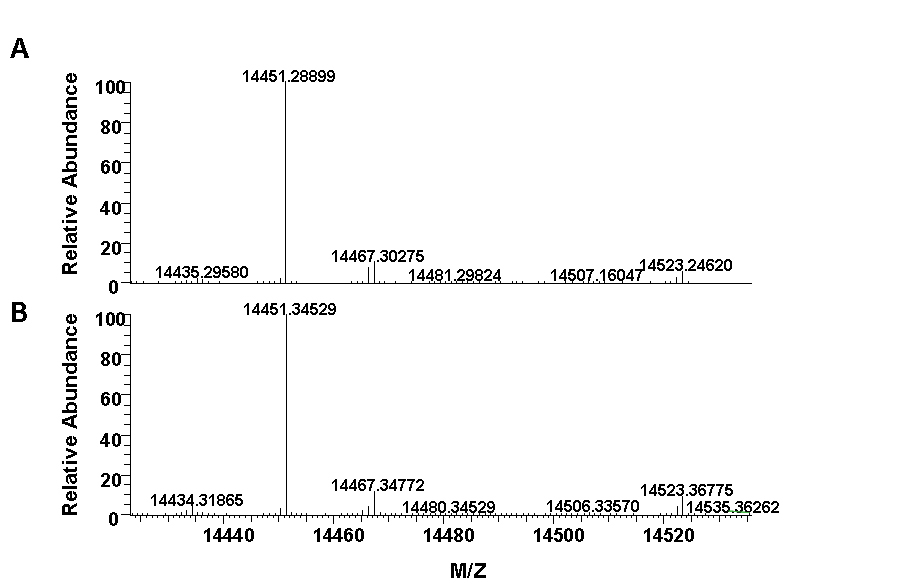

Supplement: Figure S1 — Nano-LC-ESI-MS results showed oxidative modifications of α-synuclein were not affected by the high temperature treatment. (A) α-Synuclein without heat treatment and (B) after treatment at 80°C for 6 hr. (JPG) [file pone.0053487.s001.jpg]

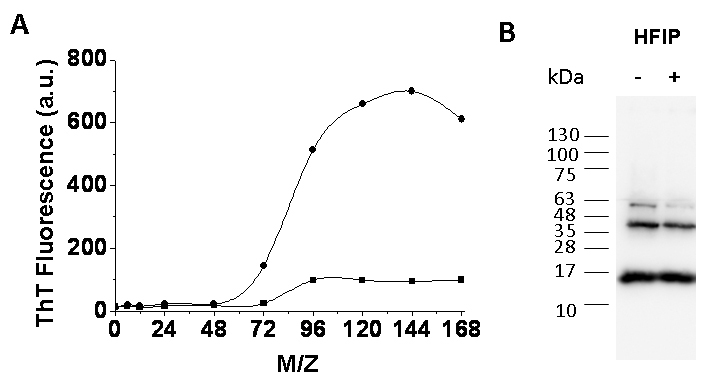

Supplement: Figure S2 — HFIP pretreatment inhibits α-synuclein fibril formation. (A) α-Synuclein samples were pretreated with (▪) and without (•) HFIP to remove pre-aggregates and dried by speed vacuum to remove the solvent. α-Synuclein was dissolved in 20 mM Tris-HCl, pH 7.4, centrifuged at 17,000×g at 4°C for 30 min and incubated in continuous shaking for 7 days. (B) PICUP assay showed α-synuclein dimer and trimer were reduced after HFIP treatment. The western blot was probed by anti-syn 211 antibody. (JPG) [file pone.0053487.s002.jpg]
