# Supplementary material for: Temperature-Dependent Structural Changes of Parkinson's Alpha-Synuclein Reveal the Role of Pre-Existing Oligomers in Alpha-Synuclein Fibrillization
Source: PLoS One. 2013 Jan 22;8(1):e53487. doi: 10.1371/journal.pone.0053487 (PMC3551866; doi:10.1371/journal.pone.0053487)
Supplement: Method S1 — Temperature effect on Parkinson's α-synuclein. (PDF) [file pone.0053487.s003.pdf]

Supplementary Information

Temperature-dependent structural changes of Parkinson's alpha-synuclein reveal the role of pre-existing oligomers in alpha-synuclein fibrillization

Winny Ariesandi[1, 2, 3], Chi-Fon Chang[1], Tseng-Erh Chen[1], and Yun-Ru Chen[1, 2]*

Supplementary Method S1

Nano-LC-ESI-MS. High resolution and high mass accuracy nanoflow LC-MS experiments were done on a LTQ Orbitrap XL ETD mass spectrometer (Thermo Fisher Scientific, San Jose, CA) equipped with a nanoelectrospray ion source (New Objective, Inc.), and accela LC system was used (Thermo Fisher Scientific, San Jose, CA). The digestion solution was injected (6 µl) at 10 µl/min flow rate on to a self packed pre-column (150 µm I.D. x 30 mm, 5 µm, 200 Å). Chromatographic separation was performed on a self packed reversed phase C18 nano-column (75 µm I.D. x 200 mm, 2.5 µm, 100 Å) using 0.1% formic acid in water as mobile phase A and 0.1% formic acid in 80% acetonitrile as mobile phase B operated at 300 nl/min flow rate. MS condition: mass range m/z 800-2000, resolution 100,000 at m/z 400. Electrospray voltage was maintained at 1.8 kV and capillary temperature was set at 200 ^{o}C.

α-Synuclein preparation by HFIP pretreatment. The lyophilized α-synuclein was pretreated with and without HFIP for 30 min to remove pre-aggregates, followed by drying in speed vacuum. α-Synuclein was dissolved in 20 mM Tris-HCl, pH 7.4, centrifuged at 17,000 x g at 4 °C for 30 min. After centrifugation, supernatant was collected and quantified by absorbance at 274 nm. The fibril formation assay was performed by addition of ThT and measuring the fluorescence with excitation at 444 nm and emission at 485 nm. α-Synuclein (25µM) was incubated at 25 °C in continuous shaking for 7 days.
